# 水产品中有害物质分析样品前处理技术研究进展

**DOI:** 10.3724/SP.J.1123.2020.07025

**Published:** 2021-01-08

**Authors:** Xingyi WANG, Yanlong CHEN, Xiaohua XIAO, Gongke LI

**Affiliations:** 1.中山大学化学学院, 广东 广州 510275; 1. School of Chemistry, Sun Yat-sen University, Guangzhou 510275, China; 2.兴义民族师范学院生物与化学学院, 贵州 兴义 562400; 2. School of Biology and Chemistry, Xingyi Normal University for Nationalities, Xingyi 562400, China

**Keywords:** 样品前处理, 环境污染物, 非法添加剂, 生物毒素, 水产品, sample preparation, environmental pollutants, illegal additive, biotoxins, aquatic products

## Abstract

水产品含有丰富的蛋白、维生素和多种微量元素,是人们摄取动物性蛋白质的重要来源之一,我国是世界上最大的水产品消费国,其质量安全问题一直备受关注。但水产样品基质复杂,有害物质的含量低,须对其进行分离富集后才能进行检测,传统的液-液萃取、固相萃取和快速固相分散萃取等样品前处理技术在水产品分析中得到广泛应用,同时针对一些挥发性和超痕量有害物质检测时,固相微萃取同样体现出巨大优势。这些样品前处理技术可以有效去除基体对分析对象的干扰,提高检测方法的灵敏度和准确度。根据目标分析物性质的不同,选择合适的样品前处理技术,是水产品中有害物质分析的关键步骤。该文以水产品中有害物的来源不同,将其分为3类:(1)水产品中环境污染物的分析;(2)养殖运输和加工过程中有害物的分析;(3)水产品中生物毒素的分析。以这3类有害物质的分析为主线,综述了近10年水产品中有害物质分析的样品前处理技术,包括液-液萃取、固相萃取、固相微萃取、快速固相分散萃取和磁性固相萃取等。此外,还对各种技术的优缺点进行了探讨,并对其未来发展方向进行了展望。

## Abstract (en)

Aquatic products, which are among the most important sources of animal protein, contain proteins, vitamins, and a variety of trace elements, thus occupying an indispensable part of a reasonable diet. China is the largest consumer market of aquatic products in the world. The quality and safety of aquatic products are closely related not only to the healthy development of the aquaculture industry, but also to people’s health. However, the presence of harmful substances has a bearing on the quality and safety of aquatic products in the overall process, including breeding, processing, storage, and transportation. These harmful substances are enriched in aquatic products and are transferred to humans via the food chain. Accurate determination of such harmful substances in aquatic product samples is imperative because of their complex matrices and extremely low concentrations. Many efficient sample preparation techniques such as liquid-liquid extraction, solid-phase extraction, and QuEChERS (quick, easy, cheap, effective, rugged, and safe method) with different configurations have been developed and widely employed for preconcentration in different matrices of aquatic products. Meanwhile, solid-phase microextraction has been demonstrated to be advantageous for some volatile and ultra-trace harmful substances. Suitable sample preparation techniques are important for effectively removing matrix interferences as well as for improving the sensitivity and accuracy of the method. It is important to develop appropriate sample preparation techniques for different target compounds in aquatic products. The harmful substances in aquatic products can be segregated into three categories according to their sources: (1) environmental pollutants in aquatic products; (2) substances acquired during aquaculture, transportation, and processing; (3) biotoxins in aquatic products. This article reviews the progress in sample pretreatment techniques for three harmful substances in aquatic products over the past decade. Various sample pretreatment techniques have been summarized and described, including liquid-liquid extraction, solid-phase extraction, solid-phase microextraction, QuEChERS, and magnetic solid-phase extraction. In addition, the merits and demerits of these techniques and future research directions are discussed. Finally, we reviewed the progress in functionalized materials for the preparation of aquatic product samples. With the increasing demand for aquatic products, quick, sensitive, and practical detection methods, such as surface-enhanced Raman scattering (SERS) are gaining importance. SERS has great potential for fast and accurate on-site detection of harmful substances in aquatic products. Several nondestructive sample pretreatment techniques have also been developed for harmful substances in aquatic products. The application and development of these techniques will guarantee the safety of aquatic products. Moreover, *in vivo* solid-phase microextraction is a potential method for aquatic product analysis. This technique integrates sampling, extraction, and enrichment into a single step, thus significantly reducing the processing time, labor, and cost. Overall, with the development and application of sophisticated materials and techniques, we can expect theoretical and practical advances in aquatic product analysis.

水产食品具有营养丰富、味道鲜美等特点,已成为人类饮食中重要营养成分的来源,但由于环境污染和水产养殖运输中的一些违规操作,水产品质量问题已成为食品安全问题中的关注热点^[1,2]^。水产品中污染物的来源主要包括环境中有害污染物的迁移^[3]^,水产品养殖运输中添加剂的违规使用^[4,5]^和水产品体内的生物毒素^[6,7,8]^,这些污染物在水产品中富集并随食物链进入人体对健康造成潜在的危害,存在着致癌、致畸的风险,因此,必须加强对水产品的质量监控和检查。水产样品基质复杂,同时水产品中待测物含量较低,导致分析检测时容易出现假阳性或假阴性结果。因此,水产品安全质量监控必须依赖强大的分析检测技术。

为了提高分析方法的准确性和检测的灵敏度,水产品中有害物质分析的样品前处理技术显得尤为重要。近十年来,各种样品前处理技术在水产品有害物质分析中得到了广泛的应用(见图1)。液-液萃取(LLE)技术具有操作简单、快速高效等优点,被广泛应用于水产品安全检测中,但传统LLE很难消除水产品中色素、脂肪和蛋白质等杂质对测定的干扰。随着样品前处理技术的发展,固相萃取(SPE)技术和快速固相分散萃取(QuEChERS)技术在水产品分析中显示出很大的优势,这类技术不仅具有操作简单和样品处理快等优点,同时还可以有效去除色素和生物大分子对检测造成的干扰。与此同时,针对水产品中一些挥发性和超痕量污染物检测时,固相微萃取(SPME)和磁性固相萃取(MSPE)技术显示出较大的优势,并在水产食品安全检测和质量监控中发挥越来越重要的作用。如何有针对性地实现对水产品中污染物分析的特异性样品前处理,是水产品中有害物质分析的核心部分。本文综述了水产品中主要污染物的样品前处理技术的研究进展,并对水产品样品检测中未来样品前处理技术的发展趋势进行了展望。

**图 1:**
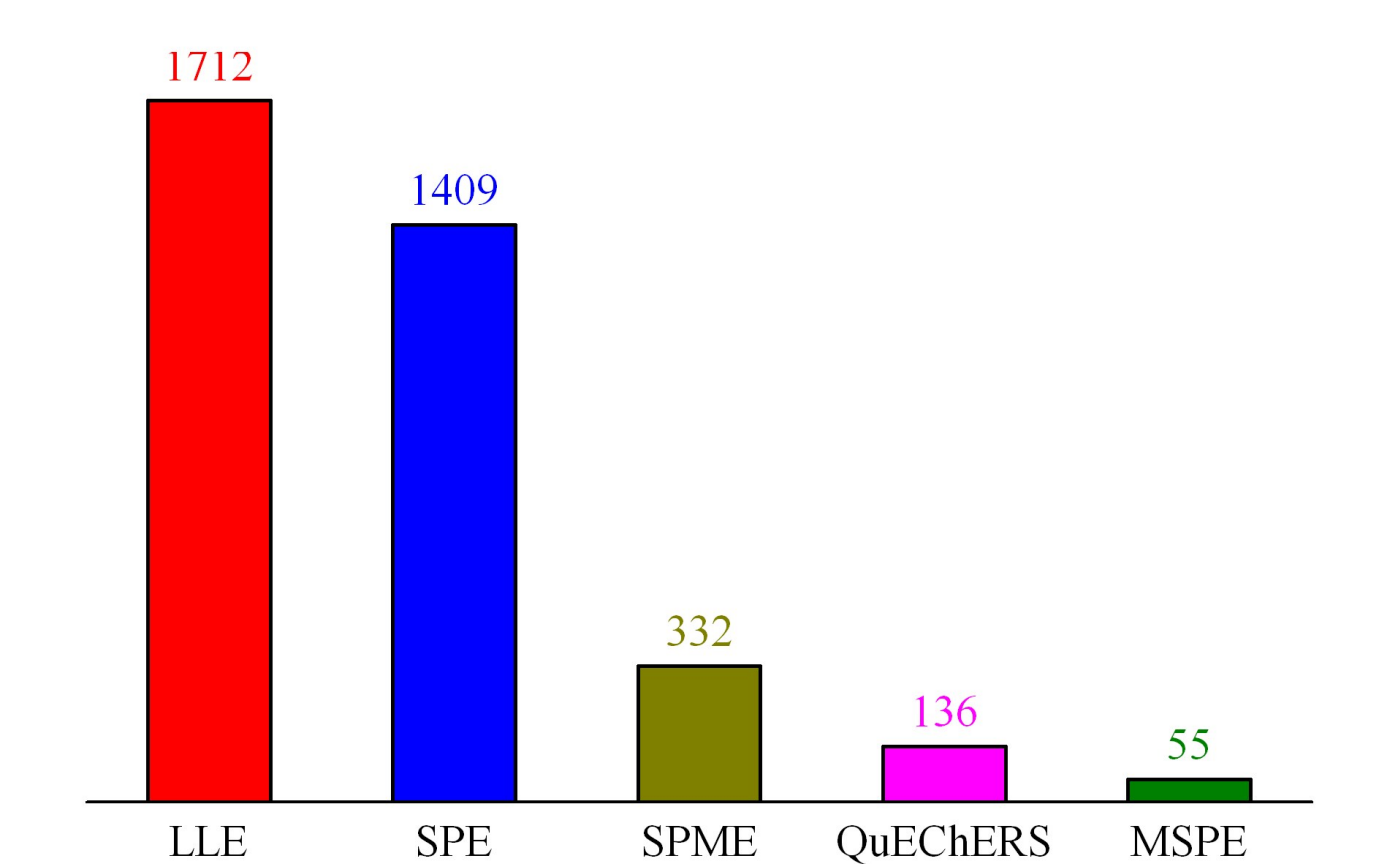
2010-2020年发表的关于水产品中有害物质分析的样品前处理技术的文献分布

## 1 水产品中的主要污染物

### 1.1 环境污染物

环境污染物如多氯联苯(PCBs)、多环芳烃(PAHs)、酚类、全氟烷基化合物(PFASs)和有机农药等是水产品中常见的有机污染物,这类有机污染物具有长期残留、不易分解和高毒性等特点,能够通过各种环境介质迁移进入水产品中,鱼和贝类是水产品中有机富集的主要生物体。这类有机污染物在水产品体内富集,经食物链到达人体后,将会对人体健康产生严重的危害^[9]^。其中,PFASs是一类新型的持久性有机污染物,其中的全氟辛基磺酸和全氟辛酸已被证实具有肝脏毒性、发育毒性、神经毒性、生殖毒性以及存在潜在的遗传和致癌性^[10]^。因此,包括我国在内的许多国家都制定出了针对食品中有机污染物的严格限量标准^[11,12]^。

随着环境污染和水产品养殖及捕捞方式的变化,水产品中重金属污染物的残留量也在不断增加,这些重金属通过食物链进入人体后,在人体中累积会损伤人体肝和肾等内脏器官,最终出现慢性中毒的现象。铅、镉、汞和砷是水产品质量监控中重点检测的4种重金属^[13]^,针对这些重金属,我国已制定了相关的限量和检测标准^[14]^。因此,为了保障水产品质量安全,对水产品体内的重金属分析检测是必不可少的。

### 1.2 养殖运输过程中的添加剂

水产品在养殖过程中通常会加入一些抗菌、杀虫和防腐物质,这些物质一旦过量和违规添加将对人体健康造成一定伤害^[15]^。其中以抗生素的违规和滥用现象最为严重,水产品中抗生素的种类比较多,常见的抗生素有喹诺酮类^[16,17]^、磺胺类^[18,19,20]^、四环素类^[21]^、大环内酯类^[22]^和氯霉素类^[23]^等,这些抗生素的性质差异较大,为同时检测带来一定的难度。

水产品在运输过程中通常会用到一些镇静麻醉类药物,以降低运输过程中的死亡率,达到安全有效运输的目的。常用的药品有丁香酚、美托味酯、硫酸奎哪啶、2-苯氧乙醇、盐酸苯唑卡因、三氯乙醛和3-氨基苯甲酸乙酯甲基磺酸盐(MS-222)等,这些药物的过量使用同样会影响人体健康^[24]^。因此,对这些物质的分析检测也非常必要。

### 1.3 生物毒素

水产品中的生物毒素目前研究较多的有河豚毒素、贝类毒素、藻类毒素、棘刺毒素、沙蚕毒素和生物胺等,这些毒素在人体中不易分解,有致畸、致癌和致突变的潜在危害。如河豚毒素是一种强烈的神经毒素,毒力比氰化钠强1250倍^[25]^,其化学性质稳定,在食品加工过程中很难被破坏,人们误食后常因缺乏特效医治而造成死亡。相比于河豚毒素,麻痹性贝类毒素是存在范围最广、危害最大的赤潮生物毒素,这类毒素明显的特征是都含有四氢嘌呤,常见的有石房蛤毒素和新石房蛤毒素^[26]^,很多国家对贝类水产品麻痹性贝类毒素有限量标准^[11,27]^。

## 2 水产品中有害物质样品前处理技术的应用

### 2.1 水产品中环境污染物的分析

2.1.1 环境中有机污染物的检测

针对待测污染物的性质及基体情况,已有多种样品前处理方法应用于水产品中环境有机污染物的选择性萃取和富集,并发展了与之对应的分析检测方法。

PCBs是一类广泛存在于环境中、持久性有机污染物,具有高毒、难降解、亲脂性强和生物富集等特点。史永富等^[28]^采用LLE-GC-MS/MS(气相色谱-串联质谱)鉴别了水产品中的7种羟基多氯联苯,回收率在70%以上。液-液萃取法是传统的样品前处理技术,该法快速、简单,但存在样品中色素、油脂和蛋白质基质干扰大等问题。江丰等^[29]^采用加速溶剂萃取-气相色谱-质谱法(ASE-GC-MS)同时测定了草鱼和海鲈鱼中32种PCBs的含量,方法回收率为71.9%~119.0%。ASE具有萃取效率高、溶剂用量少和耗时短等优点,通过ASE萃取技术,可去除水产品中油脂等杂质对检测的干扰,。针对水产品中PCBs的检测,SPME技术显示出更大的优势,可有效避免水产样品中油脂和蛋白的影响,同时提高检测的灵敏度。Guo等^[30]^利用原位生长法在不锈钢纤维上生成共价有机骨架聚合物(COF)SPME涂层(见图2),建立了SPME-GC-MS/MS测定水产品中痕量PCBs的方法,方法检出限为0.07~0.35 ng/L,回收率为87.1%~99.7%。此外,MSPE技术在萃取水产品中的PCBs也显示出良好的优势,MSPE技术的引入可缩短样品前处理时间,同时能有效地避免SPE柱堵塞的问题。Lin等^[31]^采用溶剂热法制备了一种磁性金属有机骨架(Fe_3_O_4_-MOF-5(Fe))复合材料,建立了MSPE-GC-MS方法用于萃取和富集鱼类样品6种PCBs,该方法的富集倍数为50~100倍,回收率为94.3%~97.5%。

**图 2:**
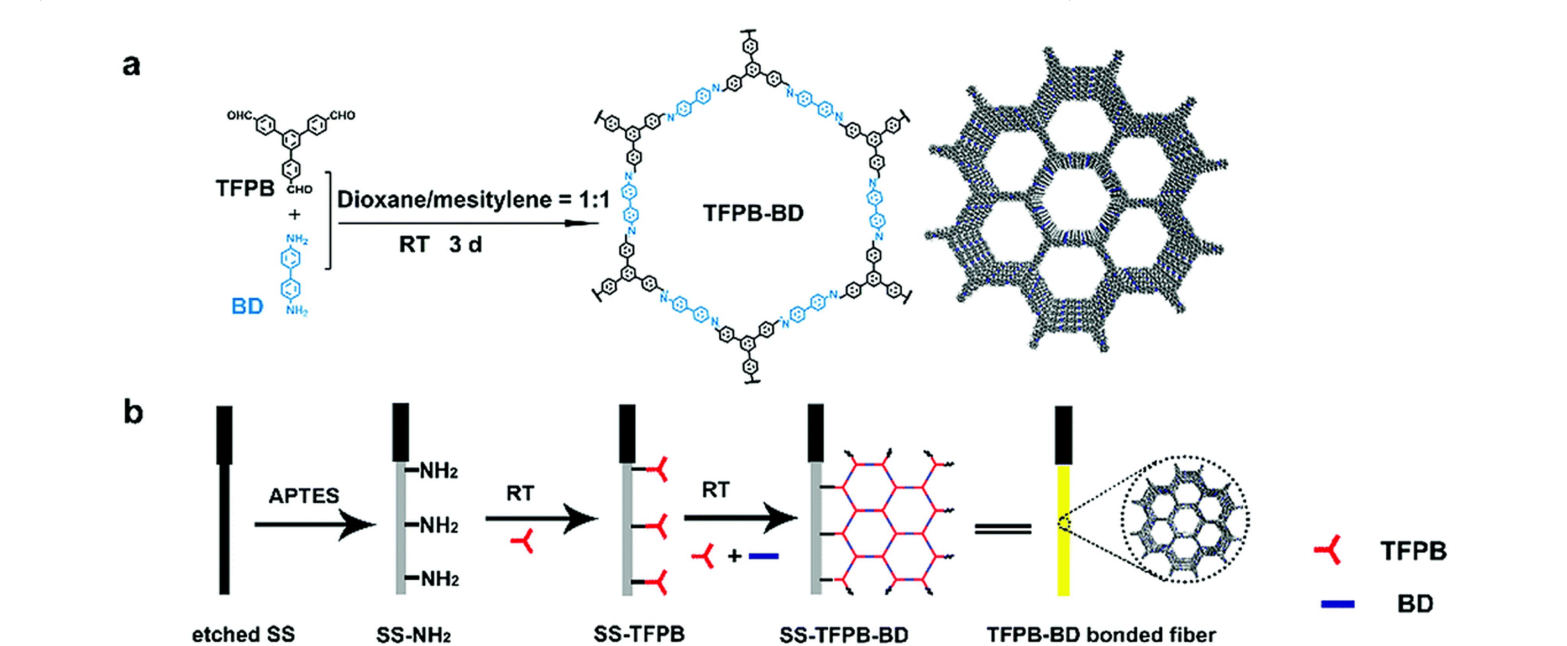
SPME涂层制备原理图^[30]^

PAHs在水产品中广泛存在,国内外已有很多针对水产品中PAHs检测的样品前处理技术研究的文献报道。张倩等^[32]^利用超声溶剂萃取结合气相色谱-质谱联用法(ULLE-GC-MS)同时测定鱼肉样品中16种PAHs,超声溶剂萃取法可快速萃取鱼肉样品中PAHs,但对鱼肉样品中的脂肪、蛋白质及其他杂质去除效果较差。刘伶俐等^[33]^建立了一种微波消解-基质固相分散萃取(QuEChERS)结合HPLC-FLD法检测虾和蟹不同组织中16种PAHs的检测方法,通过微波萃取快速地初步萃取虾蟹样品中PAHs,再利用QuEChERS实现虾和蟹样品中蛋白质、不饱和脂肪酸、色素等大分子干扰物的去除,从而提高了方法的准确性。相对于QuEChERS处理方法,SPE萃取技术在水产品PAHs萃取中显示出较大优势。杨丹丹等^[34]^建立了SPE-HPLC测定贝类体内16种PAHs的检测方法,文中指出硅酸镁-分子印迹柱(Florisil-MIP)能有效去除贝类样品中蛋白质、脂类等杂质干扰,降低基质效应,有助于PAHs定性定量,实现贝类样品中PAHs的高效富集。但是,SPE萃取水产品中PAHs时,由于基质干扰容易导致SPE柱堵塞,严重影响分析效率和检测的准确度,利用SPME可有效克服SPE的一些不足,同时在SPME上制备一些特异性吸附PAHs涂层的材料,可提高检测的灵敏度。Yuan等^[35]^制备了一种UiO-66/MoS_2_金属有机骨架材料涂层,建立了SPME-GC-MS方法检测鱼类样品中16种PAHs,与商品化聚二甲基硅氧烷(PDMS)、碳分子筛(CAR)和乙二烯基苯(DVB)涂层相比,这种复合涂层对PAHs具有更好的亲和力和更高的灵敏度。

酚类物质也是被广泛关注的水体污染物之一,关于其样品前处理技术的文献报道较多^[36,37,38,39]^。符昌雨等^[37]^建立了LLE-GC-MS方法,实现了水产品中10种含氯苯酚的快速检测,回收率在85.1%~104.5%之间。与LLE技术相比,SPE技术在水产样品酚类物质的萃取中更有优势。李诗言等^[38]^建立了一种SPE-HPLC-MS/MS法检测蟹类样品中五氯酚的检测方法,蟹类样品通过三乙胺碱化的乙腈水溶液初步提取,然后通过大粒径的阴离子交换进行富集,实现了蟹类样品中痕量的五氯酚的净化和富集。然而,在水产品样品前处理中SPE柱堵塞是一个很大问题,MSPE技术结合对酚类物质特异吸附的材料,可有效避免SPE柱堵塞问题,同时提高分析方法的灵敏度和准确度。Li等^[39]^制备了一种磁性共价有机骨架复合材料Fe_3_O_4_@COF(TpBD),将其用于虾类样品中6种甾体和酚类内分泌干扰物的高效吸附和富集,该法可快速萃取虾肉中的酚类化合物,回收率在89.6%~108.9%之间。

水产品中PFASs含量低,且样品基质复杂,因此这类物质萃取方法主要以QuEChERS和SPE为主。郭萌萌等^[40]^建立了QuEChERS-LC-MS/MS方法检测水产品中23种PFASs,首先通过酸化乙腈初步提取目标物,然后再采用QuEChERS技术经C_18_和石墨化炭黑净化,其中石墨化炭黑可有效去除一些色素干扰,回收率为75.6%~118.2%,该法操作简单,灵敏度高,适用于大批量水产品的PFASs快速检测。相比于QuEChERS萃取法,SPE萃取法具有更高的富集倍数,在水产样品中痕量PFASs分析中显示出更大的优势。Gao等^[41]^建立了一种SPE-UPLC-MS/MS检测鱼中21种PFASs的方法,在样品前处理过程中,采用乙腈进行样品提取,再通过MAX固相萃取柱净化和富集,实现全氟羧酸、全氟磺酸和全氟烷基醇类物质的选择性吸附,有效避免了鱼样品中其他杂质对检测的干扰。

水产品中有机农药残留种类繁多,基质干扰大。因此,建立一些快速高效、灵敏度高的水产品中农药的分析检测方法十分必要。张云青等^[42]^建立了加速溶剂同步萃取净化联合GC-MS/MS同时测定贝类中64种农药残留的方法,使提取和净化同步完成,可用于贝类水产品中多种农残的同时筛查。一些有特异性识别的SPE吸附剂在水产品农残分析方面显示出很大的优势。刘菁华等^[43]^制备了一种对拟除虫菊酯类农药具有特异识别的MIP用于SPE吸附剂,利用这种吸附剂实现了对鱼类样品中拟除虫菊酯类农药的高效吸附,该方法的检出限为16.1~26.5 ng/kg,回收率为85.3%~100.7%。对于大多数挥发性农药,SPME萃取技术不仅可以消除水产品中生物大分子对有机农药分析的干扰,还能提高农药检测的灵敏度。Zhang等^[44]^制备了金属有机聚合物(MOF)用于SPME整体柱材料,实现了对鱼类样品中痕量磺胺类化合物的高效萃取,其方法的检出限在1.3~4.7 ng/L之间。相比于Zhang等^[44]^的研究,Zhang等^[45]^制备的COF聚合物与MSPE技术结合,对虾样品中磺胺类化合物的富集效果更好,检出限低至0.2 ng/mL。

2.1.2 环境中重金属污染物的检测

在水产品重金属分析中,一般需要对样品进行消解使化合态的重金属转化成离子状态后再分析。通常水产品中重金属含量低,需进行富集以增加方法的灵敏度。乔晴等^[46]^建立了超声辅助提取,HPLC-电感耦合等离子体质谱联用技术(ICP-MS)测定水产品中汞形态的分析检测方法,超声场的引入提高了萃取效率,方法回收率为78.5%~96.8%。Pirsaheb等^[47]^采用悬浮有机液滴分散液液微萃取法(DLLME-SFO),结合石墨炉原子吸收光谱法(GFAAS),建立了一种DLLME-SFO-GFAAS检测养殖鳟鱼样品中重金属的分析方法,DLLME-SFO萃取方法简便、快速和环保,方法测定Cd和Pb的检出限分别为0.04和0.1 μg/kg。Javed等^[48]^制备了一种氧化石墨烯复合材料(GO),将其作为SPE吸附剂结合电感耦合等离子体原子发射光谱技术(ICP-OES)测定鱼样品中Pb和Cu, GO对目标金属离子超强的吸附能力和SPE技术的结合,排除了基体干扰,提高了检测灵敏度。为了提高吸附剂对金属离子的选择性,Barciela-Alonso等^[49]^制备了离子印迹聚合物(IIPs)作为SPE材料用于鱼样品中Cd和Pb的高效富集,利用电热原子吸收光谱法(ETAAS)实现了不同鱼样品中Cd和Pb的测定,SPE富集倍数为12.5倍,提高了方法灵敏度。MSPE技术通过在磁球表面修饰对金属离子具有特异性螯合作用的功能材料,如巯基等功能基团,提高对金属离子的特异性识别能力,能有效避免SPE柱堵塞问题,在水产品中重金属的分析中显示出很大的优势。Mashhadizadeh等^[50]^制备了双巯基乙酸盐型材料,用于MSPE吸附剂对鱼肉罐头中痕量金属离子进行选择性吸附,富集倍数为236~297倍,并利用ICP-OES技术实现了罐装金枪鱼中痕量金属离子的快速分析检测。Mei等^[51]^建立了一种磁性管内固相微萃取(MR/IT-SPME)-HPLC检测鱼样品中重金属离子的方法,结合磁分离和固相微萃取的双重优点实现了样品中Cu、Co和Hg的高效萃取,萃取率为67%~89%,明显高于传统IT-SPME的萃取率(47%~65%)。

综上,样品前处理技术在水产品环境污染物中的分析应用归纳见表1。

**表 1:** 用于水产品中环境污染物分析的样品前处理技术

Preparation technology	Sample	Analyte	Analytical method	LOD		Ref.
LLE	shrimp	PCBs	GC-MS	0.02-0.14	μg/L	[28]
ASE	fish	PCBs	GC-MS	0.1-0.5	ng/kg	[29]
SPME	fish, shrimp	PCBs	GC-MS	0.07-0.35	ng/L	[30]
MSPE	fish	PCBs	GC-MS	0.061-0.096	ng/g	[31]
ULLE	fish	PAHs	GC-MS	0.12-0.25	μg/kg	[32]
QuEChERS	shrimp, crab	PAHs	HPLC-FLD	0.2-2.0	μg/kg	[33]
SPE	shellfish	PAHs	HPLC-VWD/FLD	0.5	μg/kg	[34]
SPME	fish	PAHs	GC-MS	0.11-1.40	μg/kg	[35]
LLE	fish, shrimp	chlorinated phenols	GC-MS	0.78	μg/kg	[37]
SPE	shrimp, crab	pentachlorophenol	HPLC-MS	0.2	μg/kg	[38]
MSPE	shrimp	phenolic endocrine	HPLC-FLD	1.4-8.7	μg/L	[39]
QuEChERS	fish, shellfish	PFASs	LC-MS/MS	0.006-0.02	μg/kg	[40]
SPE	fish	PFASs	UPLC-MS/MS	2-120	pg/g	[41]
LLE	shellfish	Pesticide	GC-MS/MS	0.7-3.3	μg/kg	[42]
MIP-SPE	fish	pyrethroid insecticide	GC-ECD	16.1-26.5	ng/kg	[43]
SPME	fish	sulfonamides	UPLC-MS/MS	1.3-4.7	ng/L	[44]
MSPE	shrimp	sulfonamides	UPLC-VWD	0.2-1	ng/mL	[45]
LLE	fish	Hg	HPLC-ICP-MS	0.09-0.18	ng/mL	[46]
DLLME-SFO	fish	Cd, Pb	GFAAS	0.04-0.1	μg/kg	[47]
SPE	fish	Pb, Cu	ICP-OES	1.434, 0.048	μg/L	[48]
IIPs-SPE	fish	Cd, Pb	ETAAS	0.15, 0.5	μg/L	[49]
MSPE	fish	Ag, Cd, Pb, Hg, Cu	ICP-OES	0.01-0.09	ng/mL	[50]
MR/IT-SPME	fish	Cu, Co, Hg	HPLC-DAD	0.69-4.9	μg/kg	[51]

ASE: accelerated solvent extraction; ULLE: ultrasonic liquid-liquid extraction; MIP: molecularly imprinted polymer; DLLME: dispersive liquid-liquid microextraction; SFO: solidification of floating organic drop; IIPs: ionic imprinted polymers; MR/IT: magnetism reinforced/in tube. PCBs: polychlorinated biphenyls; PAHs: polycyclic aromatic hydrocarbons; PFASs: perfluorinated alkyl substances. FLD: fluorescence detector; VWD: variable wavelength detector; ECD: electrical conductivity detector; ICP: inductive coupled plasma emission spectrometry; GFAAS: graphite furnace atomic absorption spectrometry; OES: optical emission spectrometry; ETAAS: electrothermal atomic absorption spectrometry.

### 2.2 养殖运输和加工过程中有害物的分析

2.2.1 养殖过程中添加剂的检测

水产品养殖过程中为了缩短养殖周期,促进生长,经常会用到激素类添加剂。Wang等^[52]^采用动态微波辅助-液液萃取(DMAE-LLE)技术,联合LC-MS/MS方法实现了7种鱼样品中甾体激素的测定,在微波场中用乙腈和水依次萃取鱼类组织中的甾体激素,与传统LLE方法相比,该方法减少了有机溶剂的消耗,缩短了样品前处理时间,方法的检出限为0.03~0.15 ng/g,回收率在75.3%~95.4%之间。QuEChERS由于其快速、方便等优点,在水产样品激素类添加剂分析中显示出优势。陈秋华等^[53]^建立了QuEChERS-UPLC-MS方法检测水产品中16种激素的残留,在萃取过程中,利用正己烷脱脂,C_18_吸附剂净化,方法回收率为53.6%~135.2%,该法可用于水产品中多激素的快速筛查。Guedes-Alonso等^[54]^建立了基于微波辅助萃取/固相萃取-超高效液相色谱-串联质谱(MAE-SPE-UPLC-MS/MS)同时检测鱼样品中15种类固醇激素的分析方法,该法可在30 min内快速完成对类固醇激素的检测,采样MAE-SPE可提高萃取率,缩短前处理时间,但回收率较低(约56%)。

为了提高吸附剂材料对激素的选择性,Jiang等^[55]^制备了一种MIP材料,将其作为SPE吸附剂,建立分子印迹固相萃取(MISPE)与HPLC-FLD联用方法萃取鱼和虾组织中的雌激素,方法回收率为78.3%~84.5%,比非分子印迹固相萃取方法回收率(17.1%~21.0%)高。抗菌、杀虫药物残留分析也是水产品安全检查的一项重要指标。Du等^[56]^建立了LLE-HPLC-FLD测定虾组织样品中呋喃类添加剂残留量的检测方法,用乙酸乙酯提取待测物,该法成本低,回收率为87.4%~107%,检出限为0.24~0.26μg/kg,满足欧盟检测要求。此外,为了提高萃取率,一些新型萃取溶剂被引入水产样品中待测物的萃取。Wang等^[57]^采用离子液体([C_4_MIM][PF_6_])为萃取溶剂,建立了离子分散液-液微萃取(IL-DLLME),结合HPLC-DAD检测鱼肉中4种氟喹诺酮类药物残留的方法,在优化的实验条件下,方法的检出限为0.5~1.1 ng/mL,组织样品中4种药物的回收率为60.4%~96.3%。相比于LLE萃取,QuEChERS和SPE技术能有效去除脂肪和蛋白等大分子对待测物检测的干扰,在水产品中应用更为广泛。彭婕等^[58]^利用QuEChERS-UPLC-MS/MS技术建立了水产品中毒死蜱残留的检测方法,回收率为86.2%~103.6%,检出限为0.25 μg/kg。Sin等^[59]^利用苯基硼酸SPE萃取鱼肉样品中氟苯尼考胺(FFA),建立了SPE-LC-MS/MS检测鱼肉样品中FFA检测方法,回收率为89%~106%,其中鲑鱼和罗非鱼鱼肉的检出限分别为0.13 μg/kg和1.64 μg/kg。MIP因其特异性吸附,与MSPE技术结合不仅能提高目标物的吸附率,同时还实现了快速萃取的目的,Zhou等^[60]^制备了一种磁性分子印迹聚合物(MMIPs)材料,并结合MSPE-HPLC-UV检测鱼肉样品中多种大环内酯类抗生素(MACs),研制的MMIPs材料对MACs具有良好的选择性,对红霉素的吸附能力达到94.1 mg/g,方法检出限为0.015~0.2 μg/g,回收率为82.5%~113.1%。李攻科课题组^[61]^开发了CoFe_2_O_4_@Halloysite nanotubes/Au nanoparticle材料,用于磁性固相微萃取表面增强拉曼散射(MSPE-SERS)检测鱼样品中的呋喃妥因(见图3),高效的MSPE技术使样品分离富集在5 min内完成,极大地简化了复杂样品的前处理过程,结合SERS快速检测方法,能在15 min内完成对目标物的检测,该方法满足实际样品现场快速检测的需要。相比于MSPE, SPME在水产品样品尤其是活体样品的抗生素分析中显示出更大的优势。Tang等^[62]^建立了一种SPME-LC-MS/MS检测河豚中5种氟喹诺酮类(FQs)药物残留的新方法,在纤维表面涂覆了一层生物相容性C18-PAN材料,测定活河豚鱼背外侧肌肉中FQs残留,方法检出限为0.3~1.5 ng/g,该方法为活体水产品中FQs残留分析提供了参考。

**图 3:**
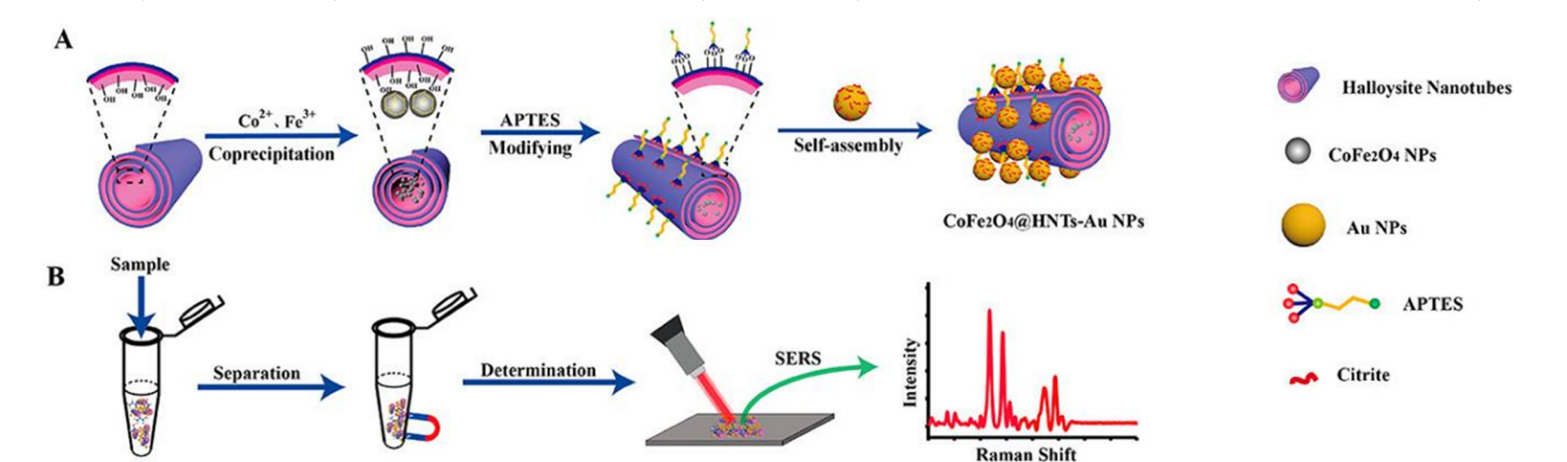
CoFe_2_O_4_@HNTs/AuNPs快速磁性固相萃取表面增强拉曼散射方法在复杂样品中的应用^[61]^

2.2.2 运输过程中添加剂的检测

为降低水产品在运输过程中的死亡率,通常添加一些镇静、麻醉类药物在水产品中,减少经济损失。常用的麻醉类药品有丁香酚、美托味酯、硫酸奎哪啶、2-苯氧乙醇、盐酸苯唑卡因、三氯乙醛和MS-222等。Botrel等^[63]^采用分散液-液微萃取法(DLLME)萃取罗非鱼组织中的薄荷醇,结合GC-MS技术实现了鱼肉组织中薄荷醇类麻醉剂的快速检测,方法检出限为0.0539 μg/L,回收率为96%~99.5%。LLE技术在水产样品麻醉类药物的萃取中很难有效去除大分子对检测的干扰,相比于LLE技术,QuEChERS和SPE技术在水产品麻醉类药物萃取中显示出较大的优势^[64,65,66]^。Li等^[64]^建立了QuEChERS-HPLC-MS/MS检测鱼类样品中MS-222的检测方法,该方法可在4 min内完成检测,检出限为2.5 μg/kg,回收率为79.6%~119.7%。SPME技术集采样、萃取和富集于一体,在很大程度上缩短了样品分析时间,尤其在活体分析中,显示出更大优势^[67,68]^。Huang等^[67]^建立了一种SPME-GC-MS快速检测活罗非鱼中5种麻醉剂残留量的方法,与传统方法相比,该方法快速(仅需10 min)、高效,可实时追踪水产品中麻醉剂的含量。水产品在运输中,还会加入保鲜剂保鲜来增加经济收入。对于非挥发性保鲜剂,LLE和SPE技术更适用这类目标物的萃取。高学慧等^[69]^利用LLE-UPLC-FLD技术建立了水产品中非挥发性保鲜剂4-己基间苯二酚的检测方法,该方法回收率为83.8%~100.7%,检出限为2.0 mg/kg。对于易挥发保鲜剂,SPME技术显示出明显优势^[70,71,72]^。Júnior等^[72]^建立了HS-SPME-GC-ECD快速分析鱼样品中三卤甲烷含量方法,该方法不使用有机溶剂,操作简单,为鱼类样品中三卤甲烷类防腐剂检测提供新的检测方法。

样品前处理技术在水产品养殖运输和加工过程中有害物的分析应用归纳如表2所述。

**表 2:** 用于水产品养殖运输过程中添加的有害物质分析的样品前处理技术

Preparation technology	Sample	Analyte	Analytical method	LOD		Ref.
MAE-LLE	fish	steroid hormones	LC-MS	0.03-0.15	ng/g	[52]
QuEChERS	fish, shrimp	hormones	UPLC-MS	0.2-2.7	μg/kg	[53]
MAE-SPE	fish	steroid hormones	UPLC-MS/MS	0.14-49.0	ng/g	[54]
MIP-SPE	fish, shrimp	estrogens	HPLC-FLD	0.023	mg/L	[55]
LLE	shrimp	nitrofuran	HPLC-FLD	0.24-0.26	μg/kg	[56]
IL-DLLME	fish	fluoroquinolones	HPLC-DAD	0.5-1.1	ng/mL	[57]
QuEChERS	fish, shrimp	chlorpyrifos	HPLC-MS/MS	0.25	μg/kg	[58]
SPE	fish	florfenicol amine	LC-MS/MS	0.13-1.64	μg/kg	[59]
MIP-MSPE	fish, shrimp	macrolide	HPLC-UV	0.015-0.2	μg/g	[60]
MSPE	fish	nitrofurantoin	SERS	0.014	mg/L	[61]
SPME	fish	fluoroquinolones	LC-MS	0.3-1.5	ng/g	[62]
DLLME	fish	menthol	GC-MS	0.0539	μg/L	[63]
QuEChERS	fish	tricaine mesylate	HPLC-MS/MS	2.5	μg/kg	[64]
SPE	fish, shrimp	diazepam	UPLC-MS	0.5	μg/kg	[65]
SPE	fish, shrimp	eugenol	UPLC-MS	0.3-0.75	μg/kg	[66]
SPME	fish	anesthetics	GC-MS	1.7-9.4	ng/g	[67]
SPME	fish	2-phenoxyethanol	HPLC-MS	0.18	μg/mL	[68]
LLE	fish	4-hexylresorcinol	UPLC-FLD	2.0	mg/kg	[69]
LLE	squid	formaldehyde	GC-MS	2.0	mg/kg	[70]
SPME	fish	formaldehyde	GC-MS	17	μg/kg	[71]
HS-SPME	fish	trihalomethanes	GC-MS	0.11-0.35	μg/kg	[72]

MAE: microwave assisted extraction; DMAE: dynamic microwave assisted extraction; IL: ionic liquid; HS: headspace; DAD: diode array detector.

2.2.3 加工过程中有害物质的检测

水产品在加工过程中也会产生一些有毒有害物质,如杂环胺类物质和苯并芘等有害物。这些有害物大多数是在高温条件下,肌肉组织样品中蛋白质发生变性而产生的^[73,74]^。Zhu等^[75]^用乙腈作为萃取溶剂,采用LLE萃取联合UPLC-MS技术建立了鱼组织样中苯并芘及其代谢产物的痕量分析。通常水产品中基质干扰大,常规的LLE萃取技术很难有效去除样品中杂质对分析检测的干扰。李攻科课题组^[76]^采用丙烯酰胺修饰石墨烯材料,结合online-MSPE萃取技术,建立了鱼组织样品中6种痕量杂环胺的分析检测方法,方法的检出限低(2.7~8.7 ng/L),能够有效去除样品中杂质对检测的干扰,准确、快速地检出鱼肉样品中痕量杂环胺。

### 2.3 水产品中生物毒素的分析

水产品中生物毒素含量低,在分析检测时受到复杂基质的干扰很大,常规LLE技术很难实现其有效萃取,SPE和QuEChERS技术更适合这类目标物的萃取^[77,78,79,80,81]^。Jansson等^[78]^利用强阳离子交换(SCX)吸附剂,建立了固相萃取与亲水性液相色谱-串联质谱法(SPE-HILIC-MS/MS)测定水产品中麻痹性贝类毒素(PST)的检测方法,改进了毒素图谱获取的方法,为法医鉴定提供依据。Wang等^[81]^以氧化石墨烯为吸附剂,建立了QuEChERS-UPLC-MS/MS检测新鲜和加工贝类产品中亲脂性海洋毒素的检测方法,石墨烯作为新型吸附剂被引入到生物毒素的萃取中,能有效去除脂类和色素等基质的干扰,回收率达到85%~117.4%。为了提高对目标分析物的特异性吸附能力,MSPE技术结合一些先进的多孔材料在水产品生物毒素分析中具有良好的前景。Zhang课题组^[82]^制备了一种MOF吸附剂材料(Fe_3_O_4_@SiO_2_@UiO-66),该MOF材料结合MSPE技术能快速高效的从复杂基质中提取软骨藻酸(DA),方法检出限低(1.45 pg/mL),回收率达到93.1%~107.3%。磁壳表面的MOF材料对软骨藻酸有很高的吸附效果,该课题组^[83]^随后又制备了一种磁性MOF复合材料(Fe_3_O_4_SPs@ZIF-8/Zn^2+^),用于贝类样品中DA的富集(见图4),该材料表面的Zn^2+^与软骨藻酸有很强的配位作用,从而提高对DA的吸附力,利用MSPE-HPLC-MS/MS技术,能在5 min内快速检测贝类样品中的DA,方法的检出限低至0.2 pg/mL,回收率为93.1%~102.3%。生物胺是鱼类在腐败菌脱氧酶作用下产生的一种毒素,这种毒素对人体有一定危害。Huang等^[84]^建立了一种SPME-GC-MS测定鱼类样品中生物胺的检测方法,该方法中SPME技术有机试剂的用量少至微升,对样品危害小,可用以目标物的原位筛查,定量限可达2.98 μg/kg,远低于美国食品药品监督管理局(FDA)规定的食品中生物胺最大允许含量50 mg/kg。河豚毒素(TTX)是一种强烈的神经毒素,毒力比氰化钠高1250倍,对人类致命剂量为2 mg^[85]^, Chen等^[86]^利用SPME-LC-MS/MS检测河豚中的内源性河豚毒素,通过静电纺丝技术制备了一种新型的PS@PDA-GA涂层,与商用PDMS和PA纤维相比,该涂层对河豚毒素具有很好的选择性,该方法的建立拓展了SPME技术的应用范围,同时也为水产品毒素分析检测提供了技术参考。

**图 4:**
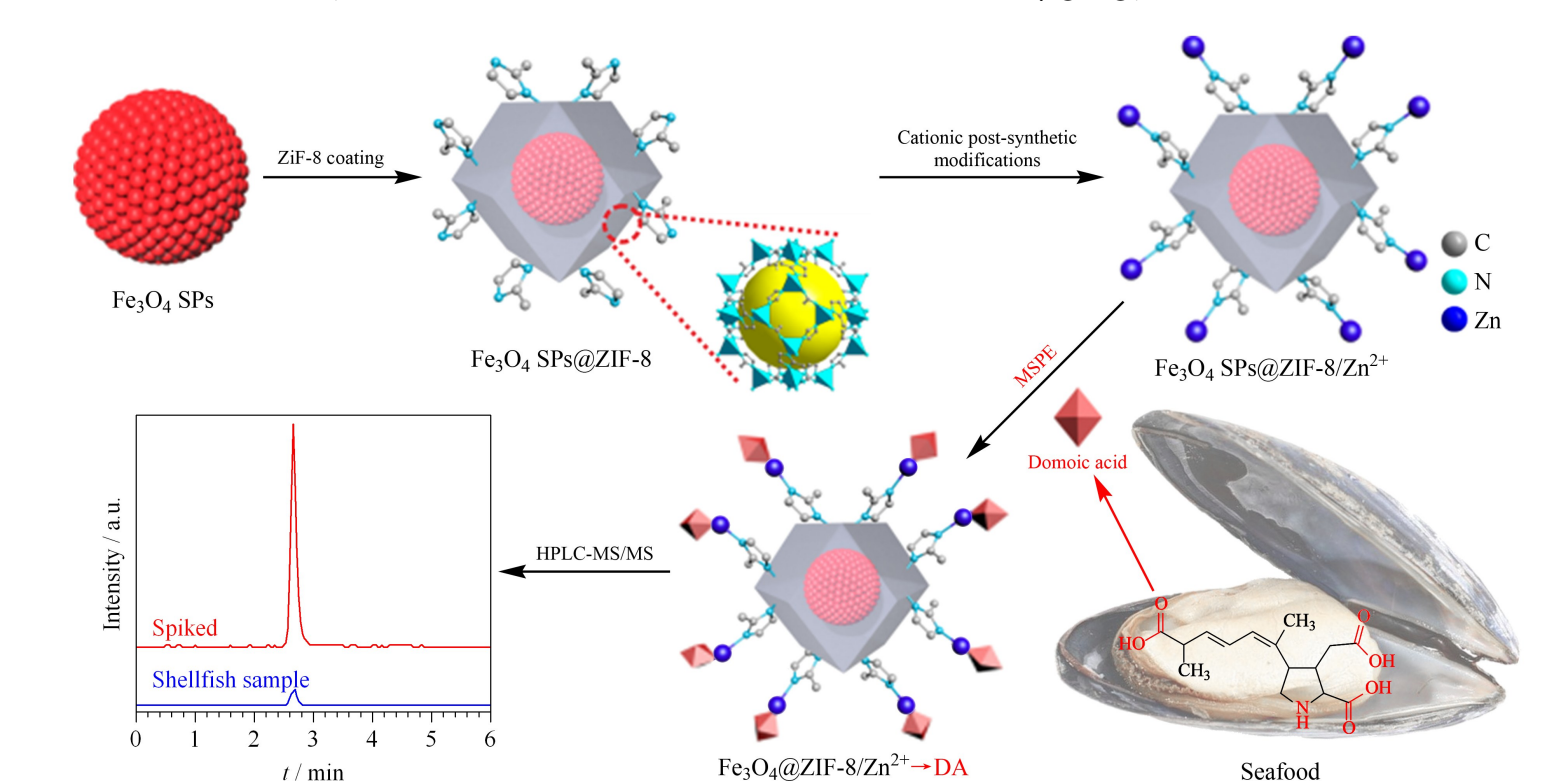
Fe_3_O_4_ SPs@ZIF-8/Zn^2+^的制备及其磁性固相萃取软骨藻酸的示意图^[83]^

综上,样品前处理技术在水产品生物毒素中的分析应用见表3。

**表 3:** 用于水产品生物毒素分析的样品前处理技术

Preparation technology	Sample	Analyte	Analytical method	LOD		Ref.
SPE	shellfish	yessotoxin	LC-MS/MS	0.15-0.30	μg/kg	[77]
SPE	shellfish	shellfish toxin	LC-MS/MS	0.1-1.1	μg/kg	[79]
QuEChERS	shellfish	shellfish toxin	UPLC-MS/MS	0.3	μg/kg	[80]
QuEChERS	shellfish	marine toxin	UPLC-MS/MS	0.10-1.47	μg/kg	[81]
MSPE	shellfish	domoic acid	HPLC-MS/MS	1.45	pg/mL	[82]
MSPE	shellfish	domoic acid	HPLC-MS/MS	0.2	pg/mL	[83]
SPME	fish	biogenic amines	GC-MS	2.98-45.3	μg/kg	[84]
SPE	pufferfish	tetrodotoxin	LC-MS/MS	2.3	ng/g	[86]

## 3 总结与展望

水产品基质复杂,基质干扰严重,待测物浓度低,因此在检测前必须要对样品进行前处理。根据水产样品和待检测目标物性质,选择合适的样品前处理方法非常重要。随着水产品中检测物种类越来越多,需要开发具有特异选择性和识别能力的新型吸附材料,以提高检测的灵敏度和准确性。一些高通量、自动化程度高的前处理联用检测技术在水产品检测领域的应用也将是今后的研究方向。随着水产样品检测数量的增多,快速样品前处理技术和快速检测技术的研究也将是水产品分析中一个很大的发展趋势,如拉曼光谱和荧光分析技术。此外,随着活体分析检测的需求,一些无损或损伤较小的样品前处理技术,如SPME也会在水产品活体分析中显示出越来越大的优势。
